# Age- and gender-related reference values of cardiac morphology and function in cardiovascular magnetic resonance

**DOI:** 10.1007/s10554-021-02160-z

**Published:** 2021-01-22

**Authors:** Johannes H. Riffel, Rebecca Mayo, Matthias Mueller-Hennessen, Evangelos Giannitsis, Hugo A. Katus, Florian Andre

**Affiliations:** 1 Department of Cardiology, Angiology and Pneumology, University of Heidelberg, Im Neuenheimer Feld 410, 69120 Heidelberg, Germany; 2 DZHK (German Centre for Cardiovascular Research), Partner Site Heidelberg, Germany

**Keywords:** Left ventricular function, Morphology, Reference values, Cardiovascular magnetic resonance

## Abstract

Cardiovascular magnetic resonance (CMR) is the reference standard for the quantitative assessment of cardiac morphology and function. The aim of the study was to determine age- and gender-related reference values for cardiac morphology and function according to current recommendations. 454 healthy volunteers (235 men, median age 52.0 (44.0–59.0) years) underwent a standard CMR scan and were divided into six groups of nearly equal size with regard to sex (male, female) and age (21–47 years, 48–57 years, 58–84 years). Left ventricular end-diastolic (LV-EDV) and end-systolic (LV-ESV) volumes and LV mass (LV-M) were measured at end-diastole and end-systole in steady-state free precession series with including papillary muscles and trabecular tissue in the LV-M. Absolute and indexed volumetric parameters were significantly different between gender groups with higher values in men compared to women (all p < 0.001). Furthermore, a significant age-dependent decline could be observed for left ventricular and right ventricular volumes (all p < 0.001), while LV-M did not show differences between the different age-groups. Parameters of longitudinal function for the left and right ventricle were higher in female compared to male subjects with a significant age-dependent decline. We provided normal values for cardiac volumes, function, and mass derived in accordance with current guidelines from a large population of healthy subjects, which can be implemented in clinical routine as a standard of reference.

## Introduction

The quantitative assessment of left ventricular (LV) and right ventricular (RV) morphology, volumes, and function is one of the most important tasks of cardiac imaging in clinical routine. These parameters are crucial for the phenotyping of patients with cardiac diseases as well as for distinguishing between healthy and diseased. Cardiovascular magnetic resonance (CMR) has hereby emerged as the non-invasive reference standard for the evaluation of cardiac morphology, volumes, and function providing accurate and reproducible measurements [1–5]. Furthermore, CMR can contribute valuable information about myocardial perfusion, edema, and fibrosis and improves risk stratification in patients with various cardiac diseases [6–9]. Normal ranges for LV morphology and function have been determined in previous CMR studies, however, results varied due to several reasons. For example, some studies included relatively small sample sizes, some did not provide values for a Caucasian population, while others did only cover a specific age span or were not conducted in strict accordance with current recommendations [10–14].

In 2013, the Society of Cardiovascular Magnetic Resonance (SCMR) published novel recommendations for post-processing in CMR. Hereby, one focus was set on the inclusion of trabecular tissue and papillary muscles in the LV mass (LV-M), on which there is still no uniformly accepted convention, yet [15].

In a previous study, we already could show that trabeculae and papillary muscles have a significant impact on the quantification of LV volumes and mass, while the inclusion of all myocardial tissue in LV-M appears advantageous. Additionally, LV-M should better be indexed to height instead of body surface area (BSA) to avoid bias in overweight subjects [16].

The aim of the present study was to provide age- and gender-specific reference values for LV and RV morphology and function derived from healthy volunteers according to the current recommendations of the SCMR.

## Materials and methods

### Study cohort

In this study, 454 subjects of a strictly selected study population of proven healthy volunteers were prospectively included. Exclusion criteria were symptoms suggestive of cardiovascular disease (dyspnea or chest pain) and past medical history of cardiovascular diseases (coronary artery disease, peripheral artery disease, carotid stenosis, heart failure, atrial fibrillation, prior pulmonary embolism, stroke or myocardial infarction, percutaneous coronary intervention or intervention of any other vessels (including bypass-surgery). Of note, hypercholesterolemia or lipid lowering medication and arterial hypertension or antihypertensive treatment were allowed in patients > 60 years. Besides that subjects were not allowed to be on any regular medication except for contraceptives or vitamins. The screening included anamnesis, physical examination, 12-lead electrocardiogram, and resting blood pressure measurement. Individuals with impaired glucose tolerance or manifest diabetes mellitus were excluded. Furthermore, in every participant, a CMR stress test (first-pass perfusion with adenosine-induced vasodilation or wall-motion analysis during dobutamine stress) was performed to exclude significant coronary artery disease. All subjects gave written informed consent. Of note, a part of the population has been part of prior studies [17, 18]. The study was approved by the local institutional ethics committee and was conducted in accordance with the Declaration of Helsinki.

### Cardiovascular magnetic resonance acquisition protocol and image analysis

Standard CMR was performed on a 1.5 T or 3 T clinical MR scanner (Ingenia Cx™ Ingenia™, Philips Healthcare, Best, The Netherlands) equipped with a cardiac phased-array receiver coil. All patients were examined in the supine position. A vector electrocardiogram was used for R-wave triggering. Short axis cine images covering the whole LV from base to apex, as well as cine long axis 2-, 3- and 4-chamber views, were obtained using a standard steady-state free precession sequence (SSFP) prior to the stress test and contrast agent application. Typical scan parameters were: repetition time (TR) 2.8 ms; echo time (TE) 1.4 ms; Flip angle 60° (1.5 T), 40° (3 T); spatial resolution 1.7 × 1.7 × 8 mm^3^ (1.5 T), 1.9 × 1.9 × 8 mm^3^ (3 T); ≥ 35 phases per cardiac cycle with a breath-hold time of 7–10 s per image and prospective gating.

All CMR scans were analyzed using certified post-processing software (cvi^42^, version 5.1.1, Circle Cardiovascular Imaging Inc., Calgary, Canada).

### LV and RV volumes

End-diastole (ED) and end-systole (ES) were defined by the phases with the largest and smallest volume of the blood pool at a mid-ventricular level. LV end-diastolic volumes (LV-EDV) and LV end-systolic volumes (LV-ESV) were derived from short-axis slices, while papillary muscles and trabecular tissue were delineated by semiautomatic threshold-selection and added to LV-M. LV volume was defined as intracavitary blood including the LV outflow tract up to the aortic valve and the LV cavity up to the mitral valve. Long axis views were used to carefully exclude aortic or atrial blood.

Corresponding to the LV, RV volumes were derived from short-axis slices and defined by endocardial contour at end-diastole and end-systole as defined by the maximal and minimal area of the blood pool in a midventricular slice. The basal slice of the RV was corroborated with the long axis views and the RV outflow tract was included in the RV volume. Trabeculae and papillary muscles were excluded from RV volume. Ejection fraction (EF) was defined as

$$EF = \left( {EDV{-}ESV} \right)/EDV.$$

### LV mass

For the assessment of LV-M, the epicardial border was traced semi-automatically and manually corrected if necessary. LV-M was calculated for end-systole as follows:

$$LV {\text{-}} M = \left( {total\;epicardial\;volume - total\;endocardial\;volume} \right) \times 1.05\;{\text{g}}/{\text{ml}}.$$

Corresponding to volumes, LV-M was determined regarding papillary muscles and trabeculae as myocardial tissue. Corresponding to the LV-EF measurements, reference views in the long axis were used to correctly identify apical and basal myocardial tissue.

LV volumes, RV volumes, and LV-M were analyzed as absolute values as well as indexed to body height.

**Fig. 1:**
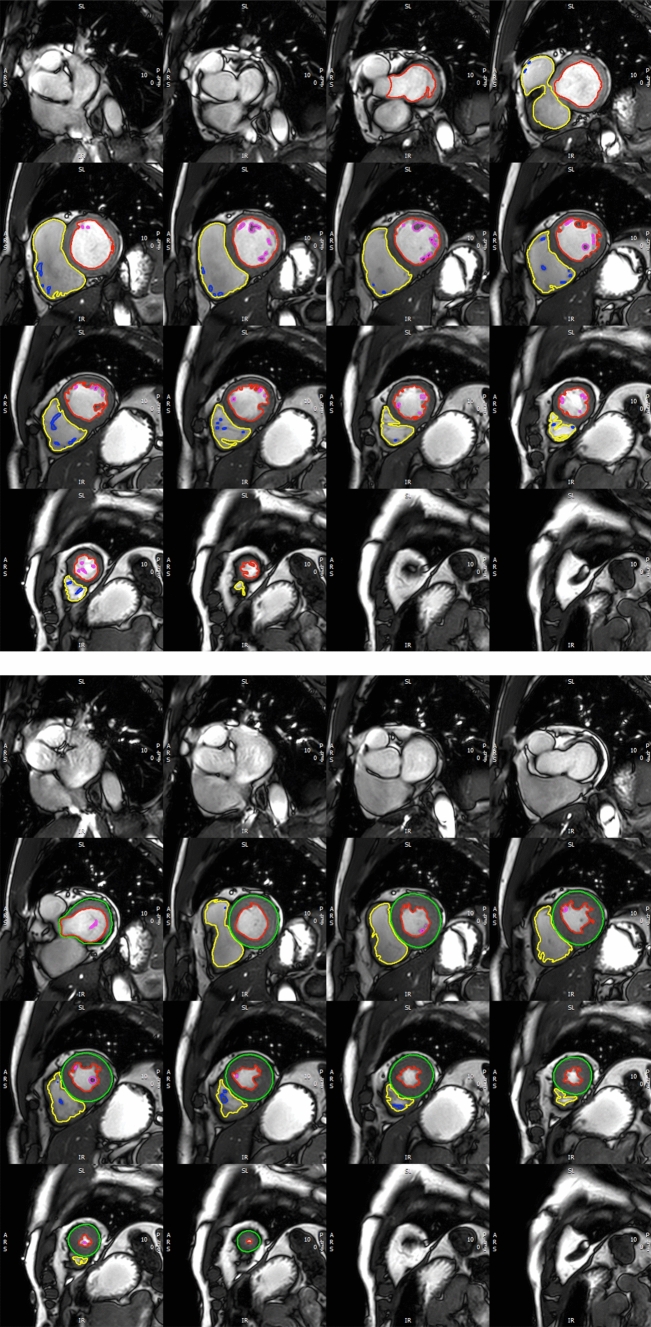
Representative example for the assessment of cardiac volumes and LV-M: Endocardial contours were delineated in end-diastole and end-systole with inclusion of the papillary muscles and trabeculae in LV-M. LV-M was assessed in end-systole

An example of the assessment of LV and RV volumes and LV-M is given in Fig. 1.

### Parameters of longitudinal function

For the assessment of longitudinal function, we measured mitral annular plane systolic excursion (MAPSE) for the LV and tricuspidal annular plane systolic excursion (TAPSE) for the RV. For this purpose, the differences between the end-diastolic and end-systolic distance between the septal mitral valve insertion and the lateral tricuspidal valve insertion, respectively, to a fixed point, where the cardiac axis intersected the chest wall, were measured.

### Standard CMR parameters of cardiac morphology

In addition to volumetric measurements, several morphological parameters were assessed in the study. Corresponding to the volumetric assessment, end-diastole and end-systole were defined by the phases with the largest and smallest volume of the blood pool at a mid-ventricular level.

LV end-diastolic diameter (LV-EDD), LV end-systolic diameter (LV-ESD), and LA diameter in ventricular systole (LA-ESD) were measured in 3-chamber views. RV end-diastolic diameter (RV-EDD) was assessed in 4-chamber views. Interventricular septal (IVS) and inferolateral wall thicknesses (LWT) were measured in SAX and cross-checked in 3-chamber views.

### Reproducibility

Inter- and intraobserver variability of the measurements of LV and RV function and LV-M were evaluated in 20 randomly selected subjects. For intra-observer variability, the same investigator (JR) performed the measurements twice within 4 weeks. For inter-observer variability, two independent blinded investigators (JR, RM) assessed the CMR examinations separately.

### Statistical analysis

Normal distribution was assessed using the D’Agostino Pearson test. As some of the data showed a non-parametric distribution, values are given uniformly as median (interquartile range, IQR). Differences between two groups were assessed using the Mann–Whitney test. More than two groups were compared applying the Kruskal–Wallis test with the posthoc analysis according to Conover and the Jonckheere-Terpstra trend test and both p-values were provided. Inter- and intraobserver variability was assessed by concordance correlation coefficient. A p-value < 0.05 was regarded as statistically significant. Statistical analysis was carried out using the software solution MedCalc (Version 19.2, MedCalc Software, Ostend, Belgium).

## Results

### Study population

The study population included 454 subjects (235 male, 219 female) with a median age of 52.0 (44.0–59.0) years. Age (51.0 (44.0–60) years vs. 52.0 (44.0–59.0) years, p = n.s.) differed not significantly between men and women, whereas men had significantly higher values for BSA (2.0 (1.9–2.1) m^2^ vs. 1.7 (1.6–1.8) m^2^, p < 0.001) and height (179.0 (175.0–183.8) cm vs. 167.0 (163.0–171.8) cm, p < 0.001).

As some of the parameters are known to be dependent on age and sex, the study population was divided into six groups of nearly equal size with regard to sex (male, female) and age (21–47 years, 48–57 years, 58–84 years).

Detailed patient characteristics are provided in Table 1.

**Table 1:** Patients characteristics

	Total (n = 454)	Men (n = 235)	Women (n = 219)	P-value
Age (years)	52.0 (44.0–59.0)	51.0 (44.0–60.0)	52.0 (44.0–59.0)	p = n.s.
Height (cm)	173.0 (167.0–180.0)	179.0 (175.0–183.8)	167.0 (163.0–171.8)	p < 0.001
Weight (kg)	73.0 (64.0–83.0)	80.0 (73.0–88.0)	64.0 (59.0–70.0)	p < 0.001
Body mass index (kg/${\text{m}}^{2}\text{)}$	24.2 (22.2–26.5)	24.9 (23.2–27.0)	23.3 (21.1–25.6)	p < 0.001
BSA (m^2^)	1.9 (1.7–2.0)	2.0 (1.9–2.1)	1.7 (1.6–1.8)	p < 0.001
Heart rate (bpm)	66.7 (60.0–74.6)	65.9 (60.0–73.2)	68.3 (62.0–75.8)	p < 0.05
Systolic BP (mmHg)	125.0 (116.0–135.0)	130.0 (120.0–136.0)	120.0 (112.3–134.8)	p < 0.001
Diastolic BP (mmHg)	78.5 (70.0–83.0)	80.0 (70.0–85.0)	75.0 (70.0–81.0)	p < 0.01
Hypertension	9	5	4	p = n.s.

### LV and RV volumes, function and LV mass (gender-related differences)

**Fig. 2:**
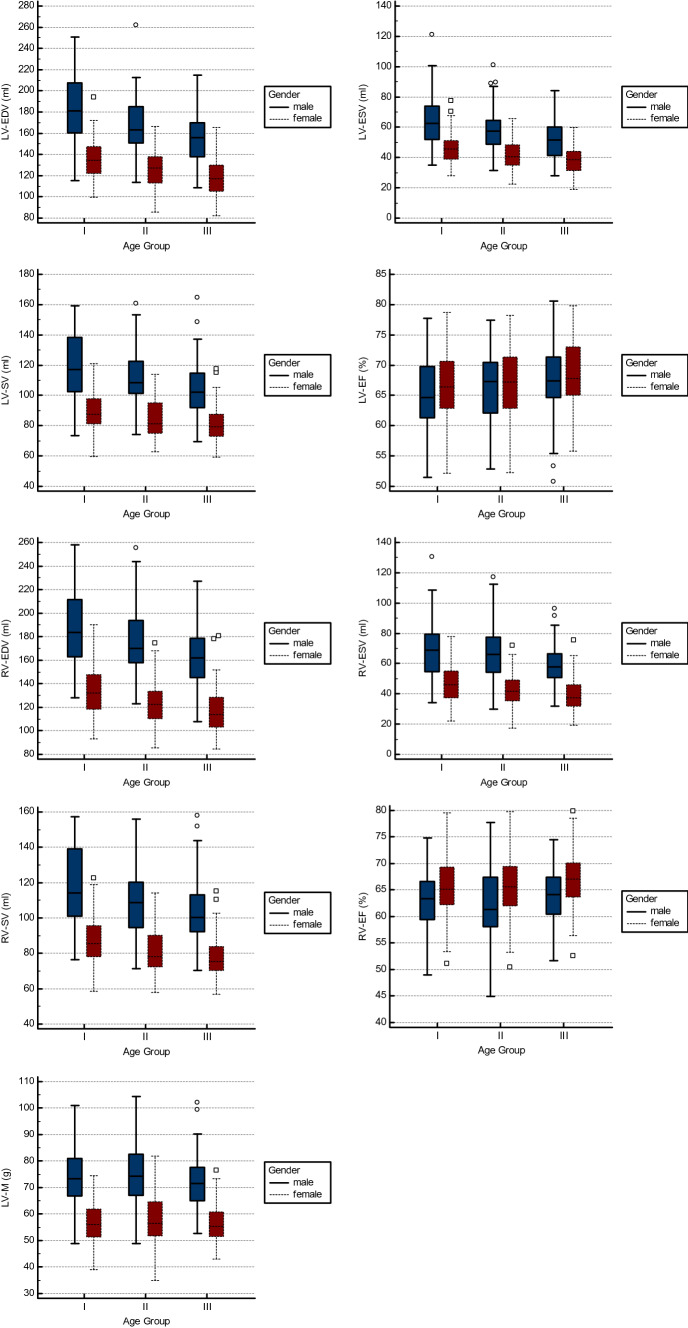
Age- and gender-related differences in LV and RV volumes, function and mass. The whiskers range from the minimum to the maximum value excluding outside values, which are shown as dots

All volumetric parameters showed significant differences between gender groups with men having higher values than women (all p < 0.001). These significant differences persisted after indexing for body height. Of note, LV-EF (66.3 (62.4–70.5) % vs. 67.2 (63.5–71.3) %, p = n.s.) did not differ significantly between men and women, while RV-EF (63.4 (59.1–67.2) % vs. 66.0 (62.9–69.5) %, p < 0.001) was significantly higher in women. Detailed results are given in Table 2 and Fig. 2.

**Table 2:** Effect of gender on cardiac morphology and function

Parameter	Men	Women	p-value
LV-EDV (ml)	165.7 (149.0–186.9)	127.3 (114.1–139.7)	p < 0.001
LV-ESV (ml)	56.5 (46.1–65.9)	41.4 (34.9–48.4)	p < 0.001
LV-SV (ml)	108.6 (98.1–125.0)	84.2 (75.6–94.5)	p < 0.001
LV-EF (%)	66.3 (62.4–70.5)	67.2 (63.5–71.3)	p = n.s.
RV-EDV (ml)	170.7 (154.6–191.2)	123.4 (108.4–137.2)	p < 0.001
RV-ESV (ml)	63.9 (53.9–74.9)	41.1 (35.1–50.1)	p < 0.001
RV-SV (ml)	108.4 (94.6–121.2)	80.5 (72.3–91.2)	p < 0.001
RV-EF (ml)	63.4 (59.1–67.2)	66.0 (62.9–69.5)	p < 0.001
LV-M (g)	146.3 (129.8–164.9)	97.0 (87.6–109.2)	p < 0.001
LV-EDVi (ml/m)	92.7 (83.4–104.2)	75.3 (69.2–83.6)	p < 0.001
LV-ESVi (ml/m)	31.4 (26.2–37.0)	24.8 (21.3–28.4)	p < 0.001
LV-SVi (ml/m)	61.1 (54.5–69.2)	49.7 (45.9–56.1)	p < 0.001
RV-EDVi (ml/m)	95.9 (86.6–106.9)	73.4 (695.9–82.0)	p < 0.001
RV-ESVi (ml/m)	35.5 (30.1–41.3)	24.9 (21.4–29.9)	p < 0.001
RV-SVi (ml/m)	60.0 (53.2–67.2)	48.1 (43.8–53.6)	p < 0.001
LV-Mi (g/m)	81.6 (73.2–90.6)	57.8 (53.6–65.3)	p < 0.001
LV-EDD (mm)	52.0 (49.0–54.0)	48.0 (45.0–51.0)	p < 0.001
LV-ESD (mm)	34.0 (31.0–36.0)	30.0 (28.0–33.0)	p < 0.001
RV-EDD (mm)	50.0 (46.0–53.0)	43.0 (41.0–46.0)	p < 0.001
LA-ESD (mm)	34.0 (30.0–38.0)	31.0 (28.0–33.8)	p < 0.001
IVS (mm)	9.0 (8.0–11.0)	7.0 (6.0–8.0)	p < 0.001
LWT (mm)	6.0 (6.0–7.0)	5.0 (5.0–6.0)	p < 0.001
MAPSE (mm)	12.0 (11.0–14.0)	13.0 (12.0–15.0)	p < 0.05
TAPSE (mm)	22.0 (19.3–24.0)	23.0 (20.0–25.0)	p < 0.05

### LV and RV volumes, function and LV mass (age-related differences)

All LV and RV volumes showed significant differences between age groups with a significant trend for an age-depended decline (all p < 0.001). Of note, LV-M 118.2 (95.1–146.2) g vs. 122.0 (97.7–155.2) g vs. 117.2 (97.8–142.6), p = n.s., p = n.s.) and LV-Mi (67.2 (55.8–82.2) g/m vs. 70.7 (57.4–86.7) g/m vs. 68.7 (59.1–81.2) g/m, p = n.s., p = n.s.) did not show significant differences between age groups. LV-EF (65.5 (61.3–70.5) % vs. 67.3 (62.2–71.2) % vs. 67.5 (64.7–71.8) %, p < 0.01, p < 0.005) increased with age, whereas the differences between age groups did not reach significance for RV-EF (64.2 (60.8–67.6) % vs. 63.9 (59.4–68.2) % vs. 65.5 (62.5–69.0) %, p = n.s., p = n.s.). Detailed results are given in Table 3 and Fig. 2.

**Table 3:** Effect of age on cardiac morphology and function

Parameter	Group I	Group II	Group III	p-value for group differences*	p-value for trend*
LV-EDV (ml)	151.2 (132.3–181.9)	146.4 (127.0–165.7)	135.5 (116.6–160.6)	p < 0.001	p < 0.001
LV-ESV (ml)	51.4 (42.7–64.7)	48.6 (38.5–59.1)	43.6 (35.4–52.8)	p < 0.001	p < 0.001
LV-SV (ml)	100.4 (86.8–118.6)	97.3 (81.1–110.1)	91.2 (77.8–105.5)	p < 0.001	p < 0.001
LV-EF (%)	65.5 (61.3–70.5)	67.3 (62.2–71.2)	67.5 (64.7–71.8)	p < 0.01	p < 0.005
RV-EDV (ml)	153.8 (130.4–186.3)	149.9 (122.7–174.1)	138.3 (114.2–163.3)	p < 0.001	p < 0.001
RV-ESV (ml)	54.6 (45.5–69.8)	51.0 (41.1–67.2)	49.7 (37.2–59.8)	p < 0.001	p < 0.001
RV-SV (ml)	97.0 (83.8–117.5)	93.9 (77.2–110.0)	89.5 (75.6–103.7)	p < 0.001	p < 0.001
RV-EF (ml)	64.2 (60.8–67.6)	63.9 (59.4–68.2)	65.5 (62.5–69.0)	p = n.s.	p = n.s.
LV-M (g)	118.2 (95.1–146.2)	122.0 (97.7–155.2)	117.2 (97.8–142.6)	p = n.s.	p = n.s.
LV-EDVi (ml/m)	88.4 (77.8–101.1)	84.5 (74.2–93.3)	79.3 (69.6–90.4)	p < 0.001	p < 0.001
LV-ESVi (ml/m)	30.0 (25.2–36.5)	27.7 (22.7–33.4)	25.4 (21.1–30.2)	p < 0.001	p < 0.001
LV-SVi (ml/m)	57.3 (51.0–67.4)	56.1 (48.5–62.0)	53.3 (46.6–60.5)	p < 0.001	p < 0.001
RV-EDVi (ml/m)	88.6 (77.1–103.4)	84.9 (73.4–97.8)	80.3 (68.5–92.2)	p < 0.001	p < 0.001
RV-ESVi (ml/m)	31.8 (26.4–38.4)	30.1 (25.3–38.4)	28.5 (22.6–34.2)	p < 0.001	p < 0.001
RV-SVi (ml/m)	56.3 (49.3–65.4)	53.7 (47.6–61.8)	52.4 (45.4–59.0)	p < 0.001	p < 0.001
LV-Mi (g/m)	67.2 (55.8–82.2)	70.7 (57.4–86.7)	68.7 (59.1–81.2)	p = n.s.	p = n.s.
LV-EDD (mm)	52.0 (48.0–54.0)	50.0 (46.0–53.0)	49.0 (46.0–52.0)	p < 0.001	p < 0.001
LV-ESD (mm)	33.0 (31.0–36.0)	32.0 (28.0–35.0)	31.0 (28.0–34.0)	p < 0.001	p < 0.001
RV-EDD (mm	45.0 (42.0–50.8)	47.0 (43.0–51.0)	47.0 (43.0–51.0)	p = n.s.	p < 0.05
LA-ESD (mm)	31.0 (29.0–34.0)	32.5 (29.0–37.0)	33.0 (30.0–38.0)	p < 0.01	p < 0.001
IVS (mm)	7.0 (7.0–9.0)	8.0 (7.0–10.0)	9.0 (8.0–11.0)	p < 0.001	p < 0.001
LWT (mm)	6.0 (5.0–6.0)	6.0 (5.0–7.0)	6.0 (5.0–7.0)	p < 0.001	p < 0.001
MAPSE (mm)	13.0 (12.0–15.0)	13.0 (12.0–14.0)	12.0 (11.0–13.0)	p < 0.001	p < 0.001
TAPSE (mm)	22.0 (20.0–25.0)	23.0 (20.0–25.0)	22.0 (19.8–24.0)	p = n.s.	p = n.s.

*The p-value for group differences was derived from the Kruskal–Wallis test and the p-value for trend from the Jonkheere-Terpstra test for trend

### Parameters of longitudinal function

MAPSE (12.0 (11.0–14.0) mm vs. 13.0 (12.0–15.0) mm, p < 0.05) and TAPSE (22.0 (19.3–24.0) mm vs. 23.0 (20.0–25.0) mm, p < 0.05) were significantly lower in men than in women.

MAPSE (13.0 (12.0–15.0) mm vs. 13.0 (12.0–14.0) mm vs. 12.0 (11.0–13.0) mm, p < 0.001, p < 0.001) showed significant differences between age groups with a significant trend for age-dependent decline, whereas the differences between age groups were not significant for TAPSE (22.0 (20.0–25.0) mm vs. 23.0 (20.0–25.0) mm vs. 22.0 (19.8–24.0) mm, p = n.s., p = n.s.).

### Standard CMR parameters of cardiac morphology

LV, RV, and LA diameters, as well as myocardial wall thicknesses, were significantly larger in men than in women (all p < 0.001, Table 2).

**Fig. 3:**
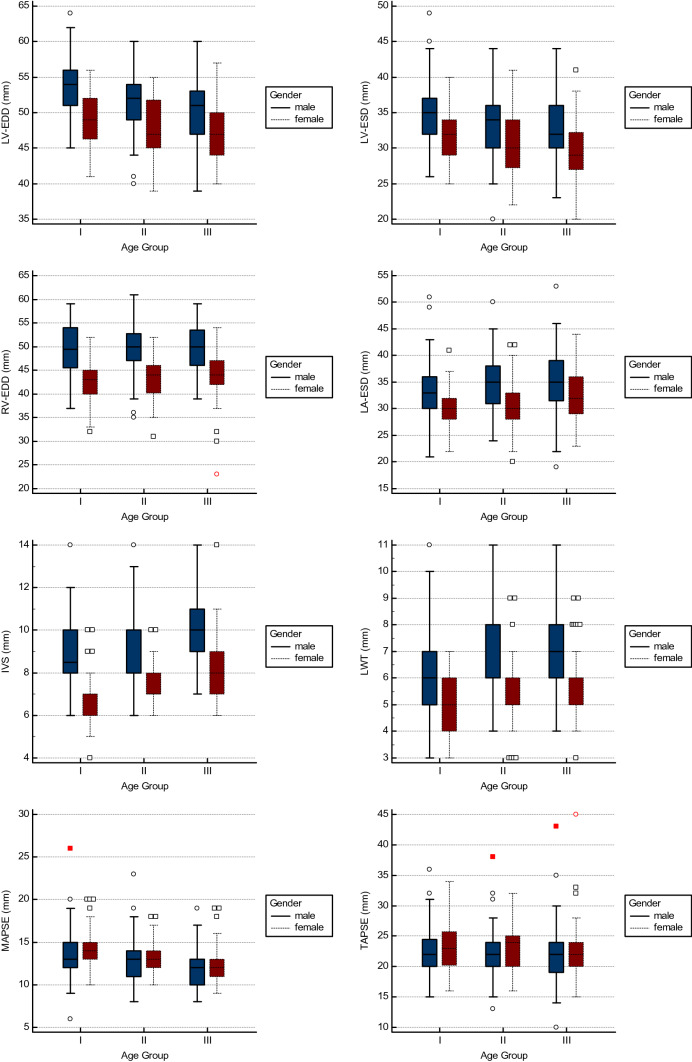
Age- and gender-related differences in morphology and longitudinal function. The whiskers range from the minimum to the maximum value excluding outside values, which are shown as dots

With increasing age, LV-EDD and LV-ESD declined (all p < 0.001). RV-EDD (45.0 (42.0–50.8) mm vs. 47.0 (43.0–51.0) mm vs. 47.0 (43.0–51.0) mm, p = n.s., p < 0.05) showed a trend for an age-dependent increase, however, the differences between age groups did not reach statistical significance. IVS (7.0 (7.0–9.0) mm vs. 8.0 (7.0–10.0) mm vs. 9.0 (8.0–11.0) mm, p < 0.001, p < 0.001) and LWT (6.0 (5.0–6.0) mm vs. 6.0 (5.0–7.0) mm vs. 6.0 (5.0–7.0) mm, p < 0.05, p < 0.001) showed a significant difference between age groups with a significant trend for an age-dependent increase. Detailed results are given in Table 3 and Fig. 3.

Of note, age- and gender-related reference values of all assessed parameters are provided in Tables 4 and 5.

**Table 4:** Age and gender-related reference values for cardiac morphology function in men

	Group I				Group II				Group III			
	Mean	SD	Median	IQR	Mean	SD	Median	IQR	Mean	SD	Median	IQR
LV-EDV (ml)	184.0	31.1	181.1	160.1–207.5	168.1	23.4	163.2	150.9–184.9	156.1	24.5	155.7	137.8–169.7
LV-ESV (ml)	64.5	17.0	62.7	52.0–74.1	57.3	14.1	57.4	48.7–64.4	51.4	12.6	51.4	41.2–60.0
LV-SV (ml)	119.5	22.3	117.1	102.6–138.4	110.8	17.0	108.6	101.4–122.6	104.7	17.8	102.2	92.1–114.6
LV-EF (%)	65.0	6.3	64.7	61.3–69.7	66.1	6.2	67.3	62.1–70.5	67.2	5.6	67.4	64.6–71.4
RV-EDV (ml)	186.9	31.8	183.6	163.1–211.4	176.0	26.6	170.1	158.0–193.7	161.4	24.9	161.9	145.3–178.4
RV-ESV (ml)	69.5	16.9	68.9	54.6–79.5	67.1	16.4	66.0	54.2–77.6	58.5	13.1	58.0	50.8–66.4
RV-SV (ml)	117.4	21.8	114.2	100.9–139.2	108.9	18.0	108.7	94.5–120.4	102.8	16.9	100.3	92.3–113.2
RV-EF (%)	62.9	5.7	63.4	59.5–66.6	62.0	6.3	61.3	58.1–67.4	63.8	5.1	64.2	60.4–67.4
LV-M (g)	149.3	29.2	146.2	130.7–171.0	150.8	23.7	154.9	130.2–166.7	141.2	22.7	141.2	127.6–151.2
LV-EDVi (ml/m)	101.1	14.7	100.0	91.3–113.5	93.8	12.8	90.9	84.9–103.4	88.2	13.4	88.0	78.4–96.4
LV-ESVi (ml/m)	35.4	8.7	34.5	29.1–40.5	32.0	8.0	31.1	27.2–36.8	29.0	7.2	28.9	23.6–33.7
LV-SVi (ml/m)	65.7	11.0	65.1	57.2–73.7	61.8	9.2	61.0	56.0–67.6	59.1	9.6	58.4	53.1–65.3
RV-EDVi (ml/m)	102.8	15.4	101.2	91.4–115.8	98.2	14.3	94.9	88.4–108.0	91.1	13.3	90.5	82.2–99.1
RV-ESVi (ml/m)	38.2	8.7	37.9	30.7–44.0	37.4	9.0	37.0	30.3–43.0	33.1	7.2	33.2	28.8–36.9
RV-SVi (ml/m)	64.6	10.8	64.0	57.0–74.6	60.7	9.7	60.7	53.1–65.5	58.1	9.1	57.7	52.4–63.5
LV-Mi (g/m)	82.0	14.5	81.7	73.9–92.6	84.1	12.9	86.5	73.6- 93.3	79.7	12.0	80.4	72.9–85.8
LV-EDD (mm)	53.6	3.8	54.0	51.0–56.0	51.5	4.3	52.0	49.0–54.0	50.1	4.0	51.0	47.0–53.0
LV-ESD (mm)	35.1	3.9	35.0	32.0–37.0	33.1	4.5	34.0	30.0–36.0	32.2	4.2	32.0	30.0–36.0
RV-EDD (mm)	49.3	5.2	49.5	45.5–54.0	50.1	5.5	50.0	47.0–52.8	50.0	5.1	50.0	46.0–53.5
LA-ESD (mm)	33.3	5.2	33.0	30.0–36.0	34.7	5.2	35.0	31.0–38.0	35.1	6.2	35.0	31.5–39.0
IVS (mm)	8.8	1.6	8.5	8.0–10.0	9.6	1.5	10.0	8.0–10.0	10.2	1.7	10.0	9.0–11.0
LWT (mm)	6.4	1.5	6.0	5.0–7.0	6.6	1.6	6.0	6.0–8.0	6.9	1.5	7.0	6.0–8.0
MAPSE (mm)	13.6	2.7	13.0	12.0–15.0	12.9	2.3	13.0	11.0–14.0	12.0	2.3	12.0	10.0–13.0
TAPSE (mm)	22.3	4.1	22.0	20.0–24.5	22.0	4.1	22.0	20.0–24.0	21.9	4.7	22.0	19.0–24.0

**Table 5:** Age and gender-related reference values for cardiac morphology function in women

	Group I				Group II				Group III			
	Mean	SD	Median	IQR	Mean	SD	Median	IQR	Mean	SD	Median	IQR
LV-EDV (ml)	135.7	17.4	134.3	122.4–147.6	126.2	17.0	127.0	113.2–138.0	119.3	17.4	117.0	105.5–129.9
LV-ESV (ml)	45.8	10.1	45.8	39.0–51.3	41.7	9.1	40.4	34.9–48.2	37.8	9.1	38.5	31.3–43.9
LV-SV (ml)	89.9	13.1	87.7	81.4–97.7	84.5	13.0	81.3	74.9–95.1	81.5	12.3	79.3	72.9–87.6
LV-EF (%)	66.3	5.5	66.5	62.9–70.6	67.0	5.3	67.2	62.9–71.3	68.5	5.3	67.8	65.1–73.0
RV-EDV (ml)	133.3	20.5	132.0	118.4–148.0	123.4	20.0	122.6	110.5–133.6	116.7	20.1	113.9	103.3–128.5
RV-ESV (ml)	46.6	10.9	46.1	37.6–54.9	42.2	10.9	41.8	35.6–49.2	39.0	10.9	37.3	31.9–46.2
RV-SV (ml)	86.7	13.6	85.4	78.1–95.5	81.2	13.4	78.2	72.4–90.4	77.7	11.6	75.5	70.4–83.8
RV-EF (%)	65.2	5.3	65.1	62.3–69.3	66.1	5.9	65.6	62.0–69.4	67.0	5.0	67.1	63.7–70.1
LV-M (g)	97.9	15.2	96.1	87.3–107.0	98.5	16.8	97.8	87.6–110.2	99.2	14.5	97.3	88.3–110.0
LV-EDVi (ml/m)	80.5	9.5	78.2	73.6–87.8	76.0	9.3	75.1	69.9–83.7	71.9	9.5	70.1	65.4–79.2
LV-ESVi (ml/m)	27.1	5.7	27.0	23.1–30.5	25.2	5.4	24.5	21.4–28.5	22.8	5.4	23.3	19.0–26.6
LV-SVi (ml/m)	53.3	7.4	51.8	48.5–57.8	50.9	7.0	49.2	45.2–56.1	49.1	6.6	47.7	45.2–52.5
RV-EDVi (ml/m)	79.1	11.6	77.4	70.7–87.8	74.3	10.8	74.4	66.0–80.8	70.3	11.0	68.6	61.9–77.0
RV-ESVi (ml/m)	27.6	6.4	26.8	21.8–33.2	25.4	6.3	25.6	21.8–29.3	23.5	6.3	22.9	19.0–27.7
RV-SVi (ml/m)	51.4	7.7	50.7	46.1–56.1	48.9	7.3	48.0	43.4–53.9	46.8	6.2	46.2	43.2–49.9
LV-Mi (g/m)	58.1	8.5	56.6	52.4–64.8	59.4	9.9	57.9	53.3–67.4	59.8	8.0	59.0	54.3–64.8
LV-EDD (mm)	48.9	3.5	49.0	46.3–52.0	47.7	4.1	47.0	45.0–51.8	47.0	3.9	47.0	44.0–50.0
LV-ESD (mm)	31.6	3.4	32.0	29.0–34.0	30.4	4.2	30.0	27.3–34.0	29.3	4.1	29.0	27.0–32.3
RV-EDD (mm)	42.4	4.3	43.0	40.0–45.0	43.5	4.5	44.0	40.3–46.0	44.0	4.9	44.0	42.0–47.0
LA-ESD (mm)	30.2	3.6	30.0	28.0–32.0	30.8	4.6	30.0	28.0–33.0	32.2	4.5	32.0	29.0–36.0
IVS (mm)	6.7	1.1	7.0	6.0–7.0	7.3	1.0	7.0	7.0–8.0	8.3	1.5	8.0	7.0–9.0
LWT (mm)	4.9	1.0	5.0	4.0–6.0	5.3	1.2	5.0	5.0–6.0	5.8	1.2	6.0	5.0–6.0
MAPSE (mm)	14.0	2.2	14.0	13.0–15.0	13.3	2.0	13.0	12.0–14.0	12.2	2.2	12.0	11.0–13.0
TAPSE (mm)	23.0	3.9	23.0	20.3–25.8	23.0	3.8	24.0	20.0–25.0	22.3	4.3	22.0	20.0–24.0

## Discussion

Our study provides age- and gender-specific reference values derived from a population of healthy subjects. As CMR is currently the reference standard to measure LV and RV volumes and function with high diagnostic accuracy and reproducibility [4], specific reference values for cardiac function and morphology are mandatory to guide the clinical management of patients with cardiac disease.

### Influence of gender

All volumetric parameters, as well as LV-M, were significantly higher in male than in female participants and differences remained significant when values were indexed to height. This is in line with previously published results [10, 12, 13, 19]. In a review on CMR reference values [13], which included three studies (n = 288), absolute values for LV volumes and mass in male and female subjects were comparable to the results of our study, thereby underlining the high reproducibility of CMR measurements. Of note, trabeculae and papillary muscles were added to LV-M.

Interestingly, LV-EF did not differ significantly between both genders, while RV-EF was significantly higher in female subjects, which is also in line with previous findings [12]. However, Petersen et al. found in their study significantly higher values for LV-EF and RV-EF in females. Hereby, papillary muscles were regarded as part of the LV volume, which may be a possible explanation for the different findings regarding the LV-EF [19]. However, the clinical relevance or impact of this finding has still to be elucidated.

### Influence of age

We divided our population into six groups of nearly equal size with regard to sex and age (21–47 years, 48–57 years, 58–84 years) to analyze age-dependent differences in our study population.

We observed a significant age-related decline of LV and RV volumes with lower values in older subjects, while there was no age-dependency regarding LV-M. These findings are similar to the results of Hudsmith et al. In their study, subjects older than 35 years showed lower values for LV volumes than subjects younger than 35 years, while LV-M did not differ significantly [12]. The same has been shown by Maceira et al. in a study with 120 subjects with 10 men and 10 women in each of 6 age deciles ranging from 20 to 80 years [14].

Of note, in our study, LV-EF was significantly higher in elderly male and female subjects, whereas Hudsmith et al. reported age-dependent LV-EF changes only in men. However, the size of the different groups in this study was relatively small impairing statistical analyses [12].

Bülow et al. also observed declining values for LV-volumes with increasing age and higher values for LV-EF in older men and women in their reference population of 624 healthy subjects [20].

Regarding the RV, an inverse correlation of RV volumes with age as observed in our study is also in line with previously published literature [13].

### Standard parameters of longitudinal function

The assessment of the longitudinal function has shown to be of significant diagnostic and prognostic value in various cardiac diseases [21–23]. While strain measurements require dedicated sequences or special post-processing techniques, MAPSE and TAPSE are easily and fast measurable from standard SSFP series.

Values for MAPSE and TAPSE were significantly lower in men than in women. Furthermore, an age-related correlation with slightly higher values in the elderly could be observed for MAPSE while there was no such age-dependency for TAPSE.

In a prior study, which analyzed parameters of the longitudinal function, a significant age-dependency could be observed with lower values in the elderly for MAPSE and TAPSE, respectively [21]. However, a gender-related difference could not be detected for both parameters. Of note, the sample size was about half the size of our study. Besides, in a previous study, we could already show that parameters of longitudinal function assessed with feature tracking software were significantly higher in female compared to male healthy subjects [18]. Echocardiographic data also showed an age-and gender dependency of MAPSE and of global longitudinal strain, which is in line with our findings [24].

### Determination of normal values

To date, several studies, ranging from small populations of < 100 subjects to the UK Biobank study of Petersen et al. including 800 subjects, have determined reference values for CMR, which have been included in meta-analysis and reviews [11–14, 19, 25]. However, as the methodology and the studied underlying normal populations differed, there are still no universal reference values for CMR. In contrast to several prior trials, which also provided elaborate analyses on a reference population with a wide age span, the current study has four features and, thus, may contribute to the determination of clinical applicable reference values.

First, the study population consisted of proven healthy volunteers. The selection procedure is crucial for the composition of a reference population. While several prior studies relied mainly on the medical history and physical examination, significant coronary artery disease was ruled out by CMR stress tests in our study. Furthermore, the majority of our study population received an extended screening including physical examination, electrocardiogram, biomarker testing, and testing for diabetes. In this way, we try to ensure the cardiovascular health of our study population.

Second, different approaches for the segmentation of the LV and RV are currently employed. Especially, the attribution of the LV outflow tract to the LV volume as well as the classification of LV papillary muscles and trabeculae as LV volume or LV-M differed considerably between studies. Regarding the volumetric analysis, our approach is in contrast to the study of Petersen et al., in which papillary muscles were included as part of LV volume. In our study, we employed the current recommendation of the SCMR [15] and regarded the whole blood pool between the atrioventricular valves and the apex as ventricular volume, while LV trabeculae and papillary muscles were attributed to the LV-M.

For quantification of the RV volume, we excluded trabeculae and papillary muscles from RV volume, which is contrary to current practice in most centers and also different to the UK Biobank study of Petersen. However, corresponding to the LV all myocardial tissue should be regarded as part of myocardium. In a previous study we observed significant differences between LV stroke volumes and RV stroke volumes only when papillary muscles and trabeculae were excluded from LV-M supporting this theory [16].

In this single center study, the intra- and interreader reproducibility for volumetric analysis of LV and RV was good (Table 6). However, even when using the same definition for the LV and RV segmentation, readers from different core labs may come to varying results [26]. Thus, reference values need to be obtained from different centers using the same approach. In this way, our study may support the application of the SCMR consensus contour in clinical routine by providing thoroughly obtained normal values.

**Table 6:** Inter- and intraobserver variability (concordance correlation coefficient)

	Intraobserver	95% CI	Interoberserver	95% CI
LV-EDV	0.98	0.95–0.99	0.97	0.92–0.99
LV-ESV	0.96	0.90–0.98	0.96	0.90–0.98
LV-EF	0.9	0.78–0.96	0.91	0.80–0.96
LV-SV	0.96	0.90–0.98	0.94	0.86–0.98
RV-EDV	0.93	0.85–0.97	0.92	0.83–0.96
RV-ESV	0.91	0.82–0.96	0.92	0.81–0.97
RV-EF	0.84	0.67–0.92	0.79	0.54–0.91
RV-SV	0.88	0.73–0.95	0.82	0.62–0.92
LV-M	0.97	0.94–0.99	0.96	0.91–0.98

Beyond that, a sufficient size of the different subgroups of the study population with regard to age and gender is required to allow for statistical analyses. Our study population covered a wide age-span even surpassing the UK Biobank trial and, thus, allowed for the composition of specific subgroups of an adequate and nearly equal size enabling the assessment of age- and gender-dependent differences.

Third, in most studies, values for LV and RV parameters are indexed to BSA. However, in a prior study, we observed that myocardial hypertrophy was missed in overweight subjects when values were indexed to BSA and not to height [16]. We, therefore, indexed our values to height and not to BSA to avoid bias through pseudo-normalization in overweight subjects.

Forth, we furthermore provided easily assessable parameters for cardiac morphology and parameters of longitudinal function, which can be routinely applied into clinical practice and which should be vendor and software independent.

Several prior studies have provided normal values derived from their local population and some meta-studies have been conducted including a wider spectrum of healthy individuals. However, as our knowledge on influencing factors on normal values grows, recommendations on post-processing change and new measuring techniques are introduced into clinical routine, existing reference ranges need to be updated regularly [25].

### Limitations

All reference values were derived from a Caucasian population and may therefore not be representative of other ethnicities. Furthermore, all data has to be interpreted as cross-sectional and not longitudinal as we did not perform CMR examinations in individuals repeatedly. In our study, we did not standardize or control loading conditions, which may potentially have an impact on our measurements although the effect in healthy individuals appears to be neglectable.

Our reference values cannot be applied when papillary muscles and trabeculae were excluded from LV-M and therefore cannot be utilized in some older software tools, which do not allow the inclusion of trabecular tissue in LV-M. Furthermore, we did not compare different vendors for analysis of LV and RV volumes.

## Conclusion

CMR normal values for cardiac volumes, function, and mass derived from a large population of healthy subjects according to current guidelines are provided in our study, which can be implemented in clinical routine as reference values for a Caucasian population.

## Data Availability

The datasets used and/or analyzed during the current study are available from the corresponding author on reasonable request.

## Abbreviations

CMR: Cardiovascular magnetic resonance
LV: Left ventricular
RV: Right ventricular
LV-EDV: Left ventricular enddiastolic volume
LV-ESV: Left ventricular endsystolic volume
RV-EDV: Right ventricular enddiastolic volume
RV-ESV: Right ventricular endsystolic volume
LV-EF: Left ventricular ejection fraction
RV-EF: Right ventricular ejection fraction
LV-SV: Left ventricular stroke volume
RV-SV: Right ventricular stroke volume
LV-M: LV mass
BSA: Body surface area
SSFP: Steady-state free precession
SD: Standard deviation
IQR: Interquartile range
Fig: Figure
SCMR: Society of Cardiovascular Magnetic Resonance
TR: Repetition time
TE: Echo time
MAPSE: Mitral annular plane systolic excursion
TAPSE: Tricuspidal annular plane systolic excursion
LV-EDD: Left ventricular end-diastolic diameter
LV-ESD: Left ventricular end-systolic diameter
LA: Left atrium
LA-ESD: Left atrium diameter in ventricular systole
RV-EDD: Right ventricular end-diastolic diameter
IVS: Interventricular septal wall thickness
LWT: Inferolateral wall thicknesses
